# Acute kidney injury in Jamaicans with sickle cell disease hospitalized with COVID‐19 infection

**DOI:** 10.1002/jha2.636

**Published:** 2023-01-07

**Authors:** Lori‐Ann Fisher, Monika Asnani

**Affiliations:** ^1^ Department of Medicine The University of the West Indies Mona Kingston Jamaica; ^2^ Caribbean Institute for Health Research The University of the West Indies Mona Kingston Jamaica

**Keywords:** acute kidney injury, COVID‐19 infection, Jamaica, sickle cell disease

## Abstract

Despite a high occurrence of acute kidney injury (AKI) with COVID‐19 infection, there are no data on its incidence in sickle cell disease (SCD). We performed a single‐center retrospective chart review of persons aged >1 year with SCD, COVID‐19 infection and no prior dialysis requirement hospitalized from June 1, 2020 to May 31, 2022. Demographics, clinical, laboratory characteristics and outcomes were abstracted. AKI was defined using Kidney Disease Improving Global Outcomes (KDIGO) criteria. Of 38 patients meeting study criteria (60.6% female, mean age ± SD 38.6 ± 15.9 years), 3 (7.9%) were COVID vaccinated. Fifty‐five percent (55%) developed AKI with 7.9% (*n* = 3) requiring dialysis. Participants with AKI were older (44.9 versus 30.8 years, *p* = 0.005), with a higher proportion having baseline chronic kidney disease (52% versus 0%, *p* = 0.001). Severe COVID infection [age‐adjusted odds ratio (aOR): 8.93, 95%CI: 1.73‐45.99, *p* = 0.033], red cell transfusion (aOR 7.92, 1.47‐42.69) and decrease in hemoglobin per unit from baseline (aOR 2.85, 1.24‐2.28) were associated with AKI. Five persons died in hospital, with AKI resulting in higher median length of stay (12 versus 5 days, *p* = 0.007). Targeted COVID‐19 preventative measures and multinational longitudinal studies to ascertain the impact of AKI and COVID‐19 infection in SCD are needed.

## 1 INTRODUCTION

The Coronavirus Disease 2019 (COVID‐19) pandemic continues since its first description in December 2019. As of June 2022, there have been 528 million infections with 6.29 million deaths globally [1]. Acute kidney injury (AKI) is a common complication in hospitalized patients occurring in 30% to 80%, and is independently associated with adverse outcomes and death [2, 3, 4, 5].

Sickle cell disease (SCD) is the most common inherited hemoglobinopathy with approximately 300,000 infants born annually worldwide [6]. Jamaica, with a population of 2.9 million persons, has a high rate of SCD, accounting for 1 in 150 live births and 10% of the population with sickle cell trait (SCT) [44].

SCD leads to a multitude of clinical presentations through the life course of the individual with almost 2–3 decades reduction in lifespan [7, 8]. Renal disease is common and as populations with SCD live longer, then renal dysfunction becomes a major cause of morbidity and mortality in older individuals [9]. AKI is a common occurrence in persons with SCD. Amongst pediatric cohorts, rates of AKI using the KDIGO‐based creatinine definition ranged from 8% to 18% in hospitalizations for acute chest syndrome and painful vaso‐occulsive crises (VOC), with AKI increasing length of hospitalization and need for mechanical ventilation [10, 11, 12]. In middle‐income countries, AKI rates may be higher. The incidence of AKI in hospitalizations with VOC in Uganda was 36%, even after modifying the KDIGO classification of AKI for baseline hyperfiltration [12].

Yeruva et al., in their review of Medicaid admission data in the United States spanning 6 years, found SCD patients were three times more likely to develop AKI [13]. AKI was associated with ten times an increase in the risk of death and was an independent factor for incident CKD [13]. AKI events increase the risk of development and progression of CKD even as conversely CKD predisposes to AKI [14].

Based on data from a cohort of 2729 persons with SCT, of which 130 persons were hospitalized with COVID‐19 infection, SCT was associated with 40% increased risk of acute kidney failure compared to those without SCT and a twofold increased mortality. Furthermore, SCT was associated with premorbid chronic kidney disease. However, there was no data on SCD [45].

Patients with SCD are a vulnerable population with a high rate of multi‐comorbidity and so the emergence of the COVID‐19 infection was expected to cause severe morbidity and higher mortality among these individuals; however, the reported in hospital mortality ranged from 2% to 10% [15–17, 32]. Patients with renal dysfunction, history of stroke and those not taking hydroxyurea were at greater risk of death, whereas HbSC disease was a strong predictor of death in the French study [15, 17].

To date, we can find no report of AKI in SCD individuals with COVID‐19 infection. Our objective was to determine the prevalence of AKI among SCD patients hospitalized with COVID‐19 infection at the University Hospital of the West Indies (UHWI) in Jamaica and identify the risk factors and outcomes associated with this complication.

## 2 METHODS

### 2.1 Study population and design

This study was performed at the University Hospital of the West Indies (UHWI), which is a 579‐bed teaching tertiary hospital in Kingston, Jamaica offering both general and subspecialty in‐patient and outpatient management. Persons living with SCD also obtain primary medical care at the Sickle Cell Unit (SCU) at the Caribbean Institute for Health Research (CAHIR), University of the West Indies (UWI).

A retrospective cohort study of all SCD patients admitted to the UHWI, with a diagnosis of COVID‐19 infection from June 1, 2020 to May 31, 2022 was performed. The investigators obtained the charts from medical records department for patients admitted to the UHWI with the ICD 10 Codes for SCD and crises and COVID‐19.

Participants were included if they had documented SCD (inclusive of Hb SC, Hb SS, HbS‐beta thalassemia) in the medical record and had positive COVID‐19 reverse transcriptase‐polymerase chain reaction (RT‐PCR) on nasopharyngeal aspirate during or at the time of admission. Exclusion criteria were age under one year (due to lack of routine creatinine measurements) or preexisting End Stage Kidney disease (ESRD) or dialysis requirement prior to admission.

Ethics approval was obtained from the UWI Ethics Committee—Approval Number: CREC‐MN.241, 2021/2022. Informed consent was waived. We reviewed the outpatient records at the SCU, UWI of participants meeting study criteria. Baseline clinical data such as hemoglobin genotype, renal function (serum creatinine and urine albumin/creatinine ratio if available), lung function, hemoglobin levels, and oxygen saturation levels were extracted from the outpatient records.

Data abstracted from medical records at UHWI included patient demographics (date of birth, date of admission), baseline hemoglobin and creatinine (as defined as baseline or “well hemoglobin” from the outpatient medical records, and creatinine within 6 months from admission), admission absolute neutrophil and lymphocyte count, genotype of SCD, and admission diagnoses. Presenting clinical features (respiratory symptoms, painful crises, gastrointestinal symptoms) and physician reported chronic medical illness. CKD was defined as physician reported preexisting history of CKD (either eGFR < 60 ml/min or albuminuria >30 mg/g for greater than 3 months) documented in the in‐patient or outpatient record. Hypertension, diabetes mellitus and heart disease were similarly defined as physician or provider reported in the medical record. Chronic lung disease was defined as a preexisting lung disease (pulmonary fibrosis, bronchial asthma or chronic obstructive lung disease) documented in the physician assessment of the in‐patient and outpatient records. Acute chest syndrome, hepatic or splenic sequestration and painful crises were defined as physician or provider assessment of these diagnoses in the index admission. COVID‐19 vaccination was determined by chart review, as self‐reported “fully vaccinated” or completed the vaccine series for the COVID‐19 vaccine.

Laboratory values for renal function (serum creatinine) during admission, need for red cell or exchange transfusion during admission, medications, and lab values inclusive (serum creatinine, hemoglobin, WBC count, bilirubin, lactate dehydrogenase [LDH], D‐dimer, and ferritin) were extracted from the electronic laboratory record. Oxygen requirement, need for mechanical ventilation, dialysis requirement, length of stay, and in‐hospital death from any cause were determined from the charts. Severity of COVID‐19 infection was classified by the Centre for Disease Control (CDC) severity categories as asymptomatic, mild, moderate severe, and critical illness by clinical investigators reviewing the medical record [18].

### 2.2 Measurements

All laboratory measurements were obtained from the UHWI laboratories.

Samples for creatinine were analyzed using a Cobas c111 Analyzer (Roche). Estimated glomerular filtration rate (eGFR) was calculated using the 2021 CKD‐EPI equation [19].

Blood counts and automated differentials were obtained from output from a Cell‐Dyn Ruby‐Hematology Analyzer. Neutrophil/lymphocyte ratio was defined as the absolute neutrophil count divided by the lymphocyte count.

### 2.3 Study outcomes

Acute kidney injury was defined by Kidney Disease Improving Global Outcomes (KDIGO) criteria (for details of KDIGO Staging, see Supplemental Table S1) [19, 20].

According to these criteria, patients were assigned to KDIGO Stage 0 (no AKI), KDIGO Stage 1, 2, 3 based on peak creatinine in the admission compared to the baseline creatinine. If baseline creatinine was unavailable, the peak creatinine was compared to the admission creatinine.

Primary outcomes were AKI incidence and in‐hospital all‐cause mortality. The latter was defined as death by any cause during the hospitalization.

### 2.4 Statistical analysis

Study data were collected and managed using REDCap (Research Electronic Data Capture) electronic data capture tools hosted on the CAIHR's Health Insurance Portability and Accountability (HIPAA) compliant server [21]. Data were deidentified prior to export and analyzed using STATA software (version SE 17.1; StataCorp LP, College Station, Tex.).

The assumption for normality was assessed using skewness and kurtosis tests for continuous variables. The Fisher's exact test for proportions or *t* tests and Wilcoxon rank‐sum tests were used to assess differences in characteristics by AKI. Kruskal‐Wallis tests were performed for differences in medians in more than two groups.

Logistic regression models were also used to determine the effect of risk factors on AKI incidence. We opted to report an age‐adjusted odds ratio for explanatory variables with an a priori association or *p* < 0.2 on univariable analyses; age is independently associated with mortality and AKI in COVID‐19 and SCD [4, 5, 15, 22]. We excluded variables with collinearity with age (correlation coefficient ≥ 0.7)

## 3 RESULTS

In the study period, there were 41 admissions with sickle cell disease and COVID‐19 infection; two persons were excluded due to preexisting dialysis requirement and one was found to have sickle cell trait. A total of 38 persons met inclusion criteria.

### 3.1 Baseline characteristics

Of the cohort, 35 were adults and 3 were children, mean age 38.6 years, range 4 to 64 years, of which 60.6% were female. The most frequent genotype was hemoglobin SS occurring in 84.2%, with 29% having baseline CKD and lung disease, and a low rate of vaccination 7.9% and hydroxyurea use (10.5%). Over one‐third of patients had glomerular hyperfiltration (eGFR ≥ 140 ml/min/1.73 m^2^) and 7 (19%) had impaired kidney function (eGFR < 60 ml/min/1.73 m^2^) (for baseline characteristics, see Table 1).

**TABLE 1:** Baseline clinical and laboratory characteristics of the study population stratified by acute kidney injury (AKI)

Baseline participant characteristics	All (*n* = 38)	AKI (*n* = 21)	No AKI (*n* = 17)	*p* Value	Missing, *n* (%)
**Demographics**					
Age (years), mean ± SD	38.6 ± 15.9	44.9 ± 14.7	30.8 ± 14.0	**0.005**	
Female sex, *n* (%)	23 (60.6)	15 (71.4)	8 (47.1)	0.126	
**Baseline characteristics**					
*Hemoglobin genotype*					
HbSS, *n* (%)	32 (84.2)	19 (90.8)	13 (76.5)	0.348	
HbSC, *n* (%)	4 (10.5)	2 (9.5)	2 (11.8)	0.348	
HbS‐beta thalassemia, *n* (%)	2 (5.3)	0 (0)	2 (11.8)	0.348	
Baseline eGFR, ml/min/1.73 m^2^, mean ± SD	121.8± 53.8	108.7 ± 60	137.2 ± 41.5	0.109	1 (2.6)
Baseline hyperfiltration (eGFR ≥ 140 ml/min/1.73 m^2^), *n* (%)	14 (37.8)	6 (30.0)	8 (47.1)	0.286	1 (2.6)
Baseline impaired kidney function (eGFR < 60 ml/min/1.73 m^2^), *n* (%)	7 (18.9)	7 (100)	0	0.028	1 (2.6)
Baseline hemoglobin (g/dl), mean ± SD	7.7 ± 1.2	7.8 ± 1.0	7.7 ± 1.4	0.746	1 (2.6)
Baseline WBC (× 10^9^/L), mean ± SD	13.7 ± 3.6	13.7 ± 3.3	13.7 ± 4.1	0.984	1 (2.6)
Chronic kidney disease, *n* (%)	11 (29.0)	11 (52.4)	0 (0)	**0.001**	
Chronic lung disease, *n* (%)	11 (29.0)	10 (47.6)	1 (5.9)	**0.005**	
Hypertension, *n* (%)	11 (29.3)	10 (43.4)	1 (6.7)	**0.026**	
Hydroxyurea use, *n* (%)	4 (10.5)	3 (14.3)	1 (5.9)	0.387	
COVID‐19 vaccination, *n* (%)	3 (7.9)	3 (14.3)	0 (0)	0.158	

eGFR = estimated glomerular filtration rate, WBC = white blood cell count, NSAID = nonsteroidal anti‐inflammatory drugs, ICU = intensive care unit.

*Note*. *p* Values highlighted in bold are statistically significant (i.e., *p* ≤ 0.05).

AKI occurred in 21 (55%) of persons, with the majority of AKI, 76.2% 16, being present on admission. KDIGO Stage I AKI occurred in 21% 8, with 10.5% 4 having Stage II and Stage III AKI in 23.6% 9. Intermittent hemodialysis was required in 3 (7.9%) of persons during the hospitalization (see Table 2).

**TABLE 2:** Clinical and laboratory characteristics and outcomes of the study population stratified by acute kidney injury (AKI)

Participant characteristics	All (*n* = 38)	AKI (*n* = 21)	No AKI (*n* = 17)	*p* Value	Missing *n* (%)
Admission systolic blood pressure (mmHg), mean ± SD	115.9 ± 25.6	111.1 ± 25.4	121.5 ± 25.3	0.224	1 (2.6)
Admission respiratory rate (breaths/min), mean ± SD	25.4 ± 4.7	27.7 ± 4.8	22.4 ± 2.4	**<0.001**	1(2.6)
Admission oxygen saturation (%), median (IQR)	90.9 ± 6.9	88.1 ± 7.6	94.3 ± 4.0	**0.005**	
Admission hemoglobin (g/dl), mean ± SD	7.1 ± 2.0	6.6 ± 2.0	7.8 ± 1.7	**0.051**	
Admission WBC (× 10^9^/L), mean ± SD	15.2 ± 7.4	15.1 ± 8.5	15.4 ± 5.9	0.444	1(2.6)
NLR on admission, mean ± SD	3.9 ± 3.3	4.3 ± 3.6	3.4 ± 2.9	0.422	1(2.6)
Serum potassium (mmol/L), mean ± SD	5.0 ± 1.0	5.0 ± 0.9	4.9 ± 1.1	0.774	
Serum bicarbonate (mmol/L), mean ± SD	18.5 ± 3.6	16.9 ± 3.8	20.5 ± 2.0	**0.001**	
Serum creatinine (µmoles/L), median (IQR)	51 [33, 118]	114 [46, 259]	39 [29, 47]	**0.004**	
Total bilirubin (µmoles/L), median (IQR)	56.5 [36, 96]	61.5 (32.5, 119.5)	50 (40.5, 62)	0.638	6 (15.7)
LDH (U/L), median (IQR)	782 (611, 1313)	745 (657, 1313)	835 (545, 1391.5)	0.977	13 (34.3)
D‐Dimer (ng/ml), median (IQR)	6596 (2106, 14346)	6596 (2106, 19519)	5847 (2043, 14346)	0.980	25 (55.2)
Painful vaso‐occlusive crises, *n* (%)	31 (81.6)	16 (76.2)	15 (88.2)	0.302	
Acute chest syndrome, *n* (%)	20 (52.6)	15 (71.4)	5 (29.4)	**0.012**	
*CDC—severity of COVID illness*					
Mild illness, *n* (%)	5 (13.2)	1 (4.8)	4 (23.5)	**0.017**	
Moderate illness, *n* (%)	7 (18.4)	2 (9.5)	5 (29.4)	**0.017**	
Severe illness, *n* (%)	16 (42.1)	11 (52.4)	5 (29.4)	**0.017**	
Critical illness, *n* (%)	6 (15.8)	6 (28.6)	0 (0)	**0.017**	
NSAID use in hospital, *n* (%)	12 (34.3)	2 (11.1)	10 (58.8)	**0.003**	
Oxygen (via facemask or non‐rebreather), *n* (%)	16 (42.1)	11 (52.4)	5 (29.4)	**0.005**	
High flow nasal cannula or noninvasive ventilation, *n* (%)	4 (10.5)	4 (19.1)	0 (0)	**0.005**	
Invasive mechanical ventilation, *n* (%)	2 (5.3)	2 (9.5)	0 (0)	**0.005**	
Need for ICU admission, *n* (%)	3 (7.9)	3 (14.3)	0 (0)	0.238	
Received dexamethasone, *n* (%)	22 (57.9)	17 (81.0)	5 (29.4)	**0.001**	
Need for simple red cell transfusion, *n* (%)	17 (44.7)	14 (66.7)	3 (17.7)	**0.003**	
Need for exchange transfusion, *n* (%)	1 (2.6)	1 (4.7)	0 (0)	0.509	

eGFR = estimated glomerular filtration rate, WBC = white blood cell count, NSAID = nonsteroidal anti‐inflammatory drugs, ICU = intensive care unit, NLR = neutrophil‐lymphocyte ratio.

*Note*. *p* Values highlighted in bold are statistically significant (i.e., *p* ≤ 0.05).

### 3.2 AKI risk factors

Persons with AKI were older, with a higher rate of chronic kidney and lung disease. Hypertension was also more common in persons who developed AKI. On admission, persons with AKI had higher admission respiratory rates and lower oxygen saturations compared to those without, but no difference in systolic blood pressure. There was no difference in hemolysis markers (LDH or bilirubin), white cell count or neutrophil‐lymphocyte ratio (see Table 2).

Mean bicarbonate was low for the entire cohort, but persons with AKI had lower admission bicarbonate compared to that without; there was no differences in admission potassium between groups, however.

Baseline nonsteroidal anti‐inflammatory (NSAID) use was reported in 3 (7.9%) with one‐third of persons receiving NSAIDs in hospital. A higher proportion of those without AKI had received in‐hospital NSAIDs.

Although painful bony crisis was a common presentation of COVID‐19 infection occurring in 81.6%, there was no difference between AKI and non‐AKI groups; however, a higher proportion with AKI had acute chest syndrome compared to those without. A higher proportion of persons with AKI had severe or critical COVID infection, and required oxygen, high flow nasal cannula or mechanical ventilation. Admission to the intensive care unit occurred in 3 persons, all of which had AKI.

Over half of the group received dexamethasone therapy, with a higher proportion of patients with AKI. One person with HbSC and CKD requiring invasive mechanical ventilation received tocilizumab and remdesivir therapy. Forty‐five percent of persons required simple red cell transfusion, with a higher proportion with AKI requiring transfusion. Exchange transfusion was performed in 1 (2.6%) of persons.

**FIGURE 1:**
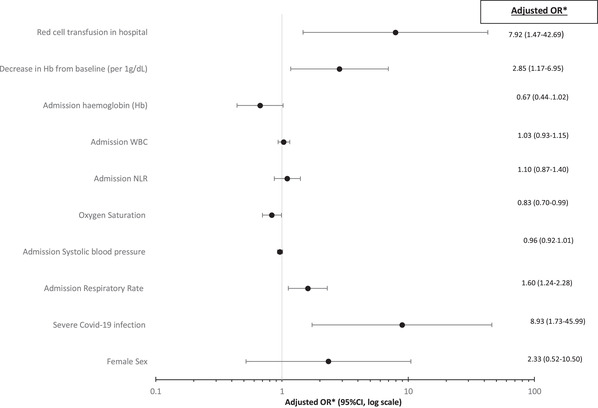
Associations between risk factors and acute kidney injury. Adjusted OR,* adjusted for age. Hb = hemoglobin, WBC = white blood count, NLR = neutrophil‐lymphocyte ratio. Error planes represent 95% Confidence Intervals

In an age‐adjusted logistic regression analyses, respiratory rate on admission [odds ratio (OR) 1.60, 95% confidence interval (CI): 1.24‐2.28, *p* = 0.009], need for red cell transfusion (OR 7.92, 95% CI: 1.47‐42.69, *p* = 0.016), severe COVID‐19 infection (CDC‐severe or critical illness) (OR 8.93, 95% CI: 1.73‐45.99, *p* = 0.033), and reduction of admission hemoglobin from baseline per 1 g/dl (OR 2.85, 95% CI: 1.17‐6.95, *p* = 0.021) were associated with AKI. Oxygen Saturation on admission (OR 0.83, 95% CI: 0.70‐0.99, *p* = 0.043) was inversely associated with AKI (Figure 1). For details of the univariable and adjusted regression results, please see Supplemental Table S2. Baseline eGFR was collinear with age (coefficient 0.70).

### 3.3 AKI outcomes

**FIGURE 2:**
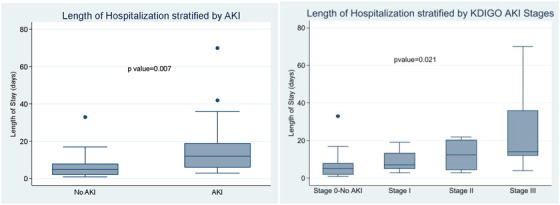
Length of hospitalization in days stratified by acute kidney injury (left) and KDIGO AKI stage (right). KDIGO = Kidney Disease Improving Global Outcomes, AKI = acute kidney injury

Median length of stay was higher in AKI versus non‐AKI (12 versus 5 days, *p* = 0.007). Length of hospitalization also increased with KDIGO Stage. Median length of stay in Stage I was 7 (IQR 5, 13.5) days, Stage II 12.5 (IQR 4.5, 20.5) days, Stage III 14 (IQR 12, 36) days, *p* = 0.021 (see Figure 2). Five persons died (13.2%), all of which had AKI.

### 3.4 Missing data

The variables with missing data are highlighted in Table 1. Persons with AKI were more likely to have missing data on LDH, but not D‐dimer or total bilirubin on admission. Please see Supplemental Table S3.

### 3.5 Pediatric outcomes

Of the 3 (all male) children of the cohort, age range 4–6 years, one developed acute kidney injury. Two had mild to moderate disease. The participant with AKI had required exchange transfusion and noninvasive ventilation. There were no deaths or renal replacement therapy.

## 4 DISCUSSION

In this single‐center study of hospitalized patients with SCD and COVID infection, we found a high rate of AKI, occurring in over half of these admissions. As expected, AKI was associated with an increased length of hospital stay. There were five deaths, all of which had AKI. There were over 2000 admissions to the COVID‐19 isolation wards at the institution, with a reported all‐cause in‐hospital mortality of 26.9% [22].

AKI in COVID‐19 infection is due to tubular dysfunction from direct viral invasion, organ crosstalk and cytokine release leading to renal hypoperfusion [23, 24, 25]. The predominant mechanism appears to be hemodynamic, with intravascular depletion and cytokine storm leading to increased capillary leak, vasodilation and myocardial injury [23, 24, 25, 26]. In the initial phases of the pandemic, the reported rate of AKI in COVID‐19 ranged from 30% to 80% [2, 3, 5, 31, 32]. Based on registry data from the United Kingdom, the incidence of AKI in hospitalized COVID‐19 patients was 31.5% [4], with approximately two‐thirds of AKI being Stage I KDIGO [4]. We found a higher rate of severe AKI (Stage II and Stage III), compared to these reports. However, there is no published comparative data on overall AKI incidence in hospitalized patients in the Jamaica or the Caribbean. The higher rate of severe AKI may represent the inherent predisposition to tubular injury in persons with SCD [12, 27, 28, 29, 30]. Hyposthenuria resulting in impaired renal adaptive response to volume depletion, renal medullary ischemia and acidosis coupled with increased risk of CKD occurring in younger age groups in SCD are likely contributing factors [27, 29].

In our study, increased respiratory rate, lower oxygen saturation, and severity of COVID infection were all associated with AKI on age‐adjusted analyses. A high proportion of persons with CKD and chronic lung disease developed AKI. These were similar risk factors identified in the other studies of AKI in COVID‐19 infection in the general population [2, 3, 4, 5, 33, 34]. Corticosteroid therapy increases risk of VOC hospitalization by fourfold in SCD [46], the etiology of which is uncertain [47, 48]. Although dexamethasone use was more frequently used in AKI participants, it is also the recommended therapy in severe COVID‐19 infection [26] and may reflect the effect of the severity COVID‐19 on the microcirculation.

NSAID use in hospital was lower in persons with AKI; however, since the majority of AKI was present at the time of admission, providers would have opted to stop or discontinue NSAIDs. Surprisingly there was a low rate of baseline NSAID use; however, this may reflect recall bias. These are for the most part over the counter medications and may not be included as home medications.

There was a higher rate of red cell transfusion and lower admission hemoglobin in AKI. Furthermore, each 1 g/dl reduction of hemoglobin from baseline resulted in a threefold increase in risk of AKI. There were no differences between bilirubin or LDH between AKI and non‐AKI groups suggesting the worsening anemia was not hemolysis mediated. Prior observational studies of SCD have also found no difference in hemolysis markers between hospitalized and nonhospitalized patients with COVID‐19 infection [12, 17]. Furthermore, Minniti et al., in their multicenter analysis of 50 hospitalized persons with COVID‐19 and SCD, found no difference in baseline or “well” versus admission hemolysis markers, despite a higher need for transfusion and lower admission hemoglobin [17]. This may suggest viral or inflammatory‐induced bone marrow suppression contributing to anemia, an effect that can occur in severe COVID‐19 infection [49]. Both anemia and red cell transfusion have been associated with increased risk of AKI in hospitalized patients [34, 35, 36], the latter due to free radical mediated tubular injury and the former from renal hypoxia [37, 38, 39]. In a study of 185 pediatric patients admitted with vaso‐occlusive crises, of which one‐third developed AKI, higher admission hemoglobin was found to be protective of AKI on univariable analyses; however, hemoglobin was not included in the multivariable analyses [12]; therefore, whether this is an independent risk factor or confounded by another factor is uncertain. That worsening anemia increased risk of AKI in COVID‐19 infection and SCD may reflect an increased inflammatory response, or viral load and may be marker of disease severity, which should be explored in further studies.

There was a low overall rate of disease modifying therapy for SCD with 11% of persons on hydroxyurea compared to 42%–55% reported in North American and European sickle cell cohorts with COVID‐19 infection [15–17, 33]. Hydroxyurea use has been associated with less severe COVID infection and reduced risk of mortality in hospitalized persons with SCD. Plausible mechanisms include hydroxyurea's effect on reducing pro‐inflammatory cytokines inclusive of interleukin‐6 (IL‐6), IL‐8 and tumor necrosis factor‐alpha (TNF‐α) and beneficial effect on the microvascular circulation though increasing nitric oxide donation and intracellular signaling in SCD [40, 41]. Although hydroxyurea is subsidized for persons with SCD since 2015 in Jamaica, its reported use is still infrequent. In advanced CKD, the use of hydroxyurea may be limited; however, our rate of CKD was lower 29% versus 44% compared to data from the United States, which reported higher hydroxyurea use [17].

There was a low rate of COVID vaccination despite most admissions occurring when vaccination was widely available. Overall vaccination reduces the risk of severe COVID infection and COVID‐related deaths [42]. At 25%, the vaccination rate in Jamaica is low, and the observed rate may reflect overall local vaccine hesitancy [43]. Targeted initiatives to improve vaccine uptake amongst persons with SCD may be needed.

This is the only study determining the incidence and outcomes of AKI in SCD in hospitalized COVID‐19 patients. However, we had a small sample size thereby limiting the precision of AKI associations and the possibility of sub‐group analyses. Urine output criteria for AKI was not used and, therefore, we may have underestimated AKI prevalence. Lastly, this was single‐center study, raising the possibility for selection bias.

AKI frequently complicates hospitalizations with SCD and COVID‐19 infection. Preventative strategies to improve vaccination adherence, studies geared toward understanding barriers to hydroxyurea use, and multinational longitudinal studies exploring the long‐term effect of COVID‐19 and AKI on SCD are needed.

## AUTHOR CONTRIBUTIONS

L‐A Fisher conceptualized the study, collected and analyzed the data, and wrote the first draft of the manuscript. M Asnani conceptualized the study and supervised the data analysis and writing of the manuscript

## FUNDING

The authors received no specific funding for this work.

## CONFLICT OF INTEREST

The authors declare they have no conflicts of interest.

## ETHICS STATEMENT

Ethics approval was obtained for this study by the Mona Campus Research Ethics Committee, The UWI. Approval Number: CREC‐MN.241, 2021/2022. Informed consent was waived.

## Supporting information

Supplemental Material

## Data Availability

The data that support the findings of this study are available from the corresponding author upon reasonable request.
