# Supplementary material for: Acute kidney injury in Jamaicans with sickle cell disease hospitalized with COVID‐19 infection
Source: EJHaem. 2023 Jan 7;4(1):37–44. doi: 10.1002/jha2.636 (PMC9928646; doi:10.1002/jha2.636)
Supplement: Supplementary file 1 — Supplemental Material [file JHA2-4-37-s001.docx]

**Supplemental Material**

| **Stage** | **Serum Creatinine Criteria** |
| --- | --- |
| I | ≥26.5 µmol/L increase from baseline  OR  1.5 -1.9X baseline creatinine |
| II | 2 to 2.9 times baseline |
| III | 3 times baseline  OR  ≥353.6 µmol/L  OR  Initiation of renal replacement therapy |

**Supplemental Table 1: Table with definitions of the creatinine-based kidney disease improving global outcomes stages of Acute Kidney Injury. Key: KDIGO (kidney disease improving global outcomes)**

| **AKI risk factors** | **Odds Ratio** | **95%CI** | **p value** | **Age-Adjusted Odds Ratio** | **95% CI** | **p value 2** |
| --- | --- | --- | --- | --- | --- | --- |
| Age | 1.07 | 1.02-1.13 | 0.011 |  |  |  |
| Female Sex | 2.81 | 0.73-10.77 | 0.131 | 2.33 | 0.52-10.50 | 0.269 |
| Oxygen Saturation on admission | 0.82 | 0.70-0.96 | 0.013 | 0.83 | 0.70-0.99 | 0.043 |
| Severe COVID-19 infection | 10.2 | 2.25-46.10 | 0.003 | 8.93 | 1.73-45.99 | 0.009 |
| Respiratory Rate on Admission | 1.59 | 1.15-2.22 | 0.005 | 1.6 | 1.12-2.28 | 0.009 |
| Systolic blood pressure on admission | 0.98 | 0.95-1.01 | 0.224 | 0.96 | 0.92-1.01 | 0.085 |
| Admission NLR | 1.09 | 0.88-1.34 | 0.413 | 1.11 | 0.87-1.40 | 0.4 |
| WBC on admission | 0.99 | 0.91-1.09 | 0.888 | 1.04 | 0.93-1.15 | 0.53 |
| Haemoglobin on admission | 0.69 | 0.47-1.02 | 0.065 | 0.64 | 0.44-1.02 | 0.064 |
| Difference in Admission Haemoglobin from baseline | 3.43 | 1.43-8.22 | 0.006 | 2.85 | 1.17-6.95 | 0.021 |
| Red cell transfusion in hospital | 9.33 | 2.00-43.62 | 0.005 | 7.92 | 1.47-42.69 | 0.016 |
| Baseline Hypertension | 10.77 | 1.21-96.21 | 0.033 | 6.23 | 0.62-63.02 | 0.121 |
| Baseline estimated GFR | 0.96 | 0.97-0.99 | 0.027 |  |  |  |

**Supplemental Table 2: Univariable and Age-Adjusted Logistic Regression for AKI. Key NLR=Neutrophil Lymphocyte Ratio. WBC= White Blood Count. P values that are in bold are statistically significant* (p<0.05)**

| **Missing Data** | **All** | **AKI** | **no AKI** | **p value** |
| --- | --- | --- | --- | --- |
| D Dimer, n (%) | 21 (55.3) | 10 (47.6) | 11(64.7) | 0.324 |
| LDH, n (%) | 13 (34.2) | 4 (19.1) | 9 (52.9) | 0.042 |
| Total Bilirubin, n (%) | 6 (15.8) | 1 (4.8) | 5 (29.4) | 0.071 |

**Supplemental Table 3: Differences in variables with missing data in AKI and non-AKI. Key LDH Lactate Dehydrogenase.**
